# Soot Erased: Catalysts and Their Mechanistic Chemistry

**DOI:** 10.3390/molecules28196884

**Published:** 2023-09-30

**Authors:** Zareen Zuhra, Shuo Li, Guanqun Xie, Xiaoxia Wang

**Affiliations:** 1 Department of Environment and Civil Engineering, Dongguan University of Technology, Dongguan 523808, China; zareenzuhraqau@gmail.com (Z.Z.); jingtao360@126.com (S.L.); wangxx@dgut.edu.cn (X.W.); 2 School of Chemistry and Materials Science, University of Science and Technology of China, Hefei 230026, China

**Keywords:** soot, impact, oxidation, catalysts, mechanism

## Abstract

Soot formation is an inevitable consequence of the combustion of carbonaceous fuels in environments rich in reducing agents. Efficient management of pollution in various contexts, such as industrial fires, vehicle engines, and similar applications, relies heavily on the subsequent oxidation of soot particles. Among the oxidizing agents employed for this purpose, oxygen, carbon dioxide, water vapor, and nitrogen dioxide have all demonstrated effectiveness. The scientific framework of this research can be elucidated through the following key aspects: (i) This review situates itself within the broader context of pollution management, emphasizing the importance of effective soot oxidation in reducing emissions and mitigating environmental impacts. (ii) The central research question of this study pertains to the identification and evaluation of catalysts for soot oxidation, with a specific emphasis on ceria-based catalysts. The formulation of this research question arises from the need to enhance our understanding of catalytic mechanisms and their application in environmental remediation. This question serves as the guiding principle that directs the research methodology. (iii) This review seeks to investigate the catalytic mechanisms involved in soot oxidation. (iv) This review highlights the efficacy of ceria-based catalysts as well as other types of catalysts in soot oxidation and elucidate the underlying mechanistic strategies. The significance of these findings is discussed in the context of pollution management and environmental sustainability. This study contributes to the advancement of knowledge in the field of catalysis and provides valuable insights for the development of effective strategies to combat air pollution, ultimately promoting a cleaner and healthier environment.

## 1. Introduction

### 1.1. Aim of Review and Distinguishing Features

The primary aim of the review is to offer a comprehensive exploration of the current state of research in the field of soot oxidation catalysts, with a particular emphasis on unraveling the mechanistic chemistry behind the catalytic process. Unlike typical reviews that provide an overview of catalysts and their applications, this review seeks to provide a deeper understanding of how catalysts, especially those based on ceria or similar materials, function at the molecular level to efficiently convert soot particles into less harmful byproducts, such as carbon dioxide and water.

The factors that distinguish this review from others in the field is its commitment to a more profound examination of the mechanistic chemistry involved in soot oxidation. It aims to uncover the precise molecular mechanisms governing the interaction between catalysts and soot particles, going beyond summarizing existing knowledge to critically assess the latest developments in the field. This in-depth analysis can be particularly valuable for researchers and engineers seeking to design more effective catalysts for emissions reduction. Furthermore, this review discusses emerging trends and challenges within the field of soot oxidation catalysis. By addressing the current limitations of existing catalysts and identifying areas that require further investigation, it can help guide future research efforts. Additionally, it adopts an interdisciplinary approach, drawing insights from various scientific disciplines, such as chemistry, materials science, engineering, and environmental science, to provide a holistic perspective on the subject matter. Lastly, the review may bridge the gap between fundamental research and practical applications by exploring how a deeper understanding of mechanistic chemistry can lead to the development of more efficient and durable catalysts. This aspect is of significant interest to industries and policymakers aiming to mitigate diesel emissions, as it can offer insights into real-world solutions for reducing the environmental impact of diesel engines. In conclusion, “Soot Erased: Catalysts and their Mechanistic Chemistry” stands out for its focus on mechanistic chemistry, in-depth analysis, consideration of emerging trends and challenges, interdisciplinary approach, and potential to provide practical solutions, making it a valuable contribution to the field of soot oxidation catalysis.

### 1.2. Soot Formation and Methodology for Soot Oxidation

A few years ago, diesel engines gained worldwide fame owing to their unique features, such as low fuel consumption, long range, and greater thermal efficiency, compared with other engines [1,2]. The other side of the picture is also quite harsh given diesel engine exhaust emissions, which create a great threat for us on this planet [3,4,5]. The composition of their exhaust emanations is a combination of different gases, vapors, particulate matter (soot), liquid aerosols, nitrogen, water, CO, NO*_x_*, SO*_x_*, and polycyclic aromatic hydrocarbons (PAHs), and they make our world alarmingly polluted with air pollution [6,7,8]. The overall composition of diesel exhausts and their threats to human life and the environment are described in Table 1 [9,10,11,12]. 

**Figure 1:**
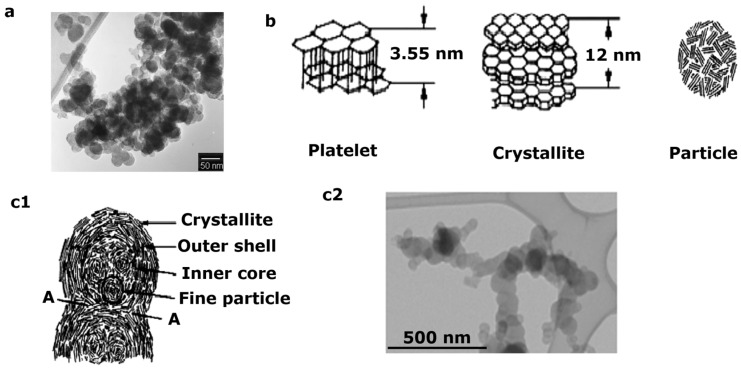
SEM image of soot aggregates in diesel exhaust (**a**), other minor structures of a soot particle (**b**), major structures of diesel soot materials (**c1**) and HR-TEM image of soot collected from a combustion chamber (**c2**) [2,22].

For these components, the major concern is the emission of soot [13]. Soot is a type of fine, black powder that is composed of carbon along with various other chemical compounds [14]. It is produced when organic matter or hydrocarbons, such as fossil fuels or wood, undergo incomplete combustion. Incomplete combustion occurs when there is not enough oxygen available for the fuel to burn completely. Soot particles are very small and can vary in size, with some being fine enough to become suspended in the air as particulate matter [15,16]. This fine particulate matter can be harmful to human health when inhaled, as it can penetrate deep into the lungs and potentially cause respiratory problems [17,18]. Moreover, soot is a common byproduct of various combustion processes, including those in car engines, industrial furnaces, and the burning of wood or other fuels in household stoves and fireplaces [19,20]. It is also a major component of air pollution and can contribute to smog formation and other environmental issues [21]. Top of Form Diesel particulates exhibit a bimodal distribution of nuclei mode particles and accumulation mixture mode particles, as shown in Figure 1 [2,22]. Nuclei mode particles are normally defined as particles having a size of less than 50 nm, and these particles easily evaporate. The accumulation of crucial carbon components where gases are adsorbed on this surface, namely, long-chain hydrocarbons, results in the formation of accumulation mode particles, which have sizes ranging from 50 to 1000 nm [23].

**Figure 2:**
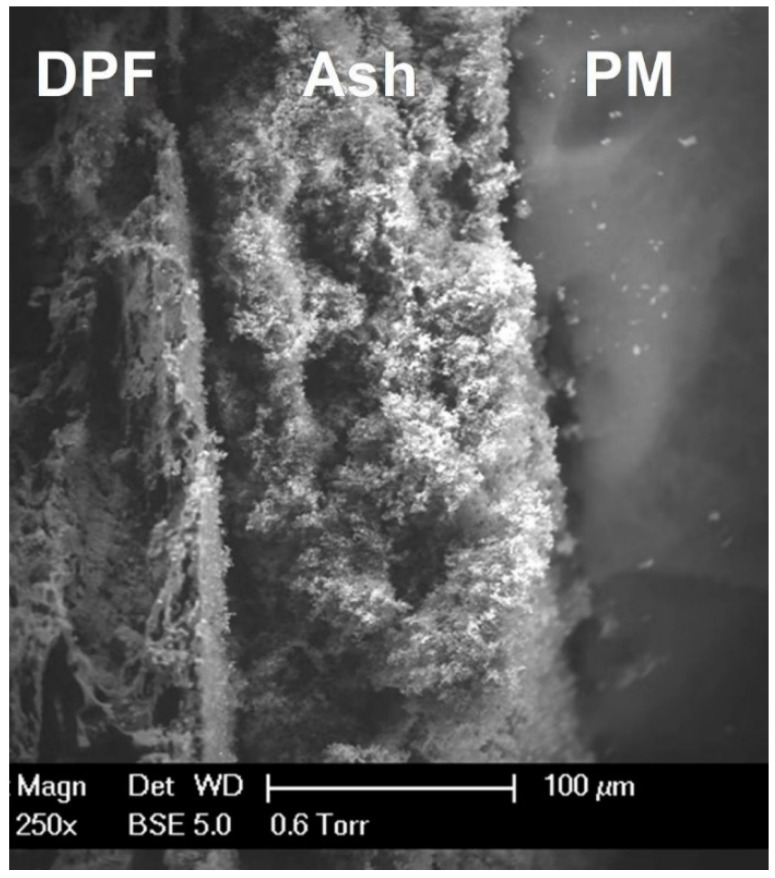
SEM images of various forms of particles [31].

These particles are initially commonly produced during the preliminary steps of soot formation and can originate from two mechanistic approaches: (1) collision coalescence, which is a physical route; and (2) surface development, which is a chemical route that occurs during the diffusion part of incineration [24]. Later, the development of the prime bits results in collisions and the subsequent formation of an agglomeration of clusters of these tiny particles; consequently, soot is formed [25]. It is well reported that the fuel undergoes two oxidation procedures that occur (1) at the fuel-rich premixed zone and (2) at the flame of diffusion on the outside boundary. PAHs are considered the focal soot pioneers and are generated in the premixed zone along with fuel-rich mixtures that develop in the middle because a portion of the surface is actually oxidized in the flame of diffusion [26,27]. The process of soot formation involves the generation of the first aromatic ring structure, the decomposition of the fuel molecule, and subsequently the growth of the polycyclic aromatic hydrocarbon (PAH), which is a molecular pioneer and particle that is used in the nucleation stages [28]. The major portion of the soot product is produced through surface development procedures that normally occur after nucleation. This phenomenon includes the connecting of gas-stage moieties, for example, these hydrocarbons, to the seeming of the elements, which allow the aggregation of the total mass while not increasing the number of soot bits [29]. These fundamental bits act as nuclei; however, weighty hydrocarbons could physically or chemically interact during the exhaust stroke or diffusion ignition. After that, the next stage is particle coagulation, wherein collisions among the particles result in an increase in the average particle size while the number of particles decreases; thus, an increase in the overall mass of the soot does not occur [30]. A scanning electron microscopic (SEM) image of various forms of black carbon particles is shown in Figure 2 [31].

**Figure 3:**
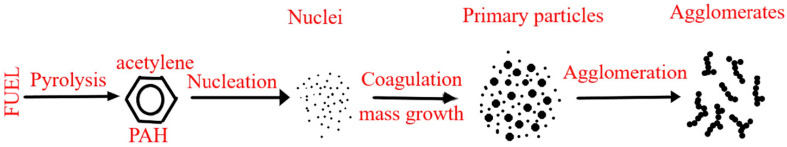
A schematic pathway for the formation of soot particles [2,31].

Nevertheless, as a result of the pyrolytic conditions that occur in the post-flame zone, graphitic carbon material is fabricated from the amorphous soot material, as revealed, resulting in a minute decrease in the mass of the particle without a change in the number of particles [17,32]. Meanwhile, PAH oxidation and soot materials are restricted as they are associated with the generation of these specific moieties [30]. A possible pathway of soot formation from the gas stage to the formation of agglomerated solid particles is illustrated in Figure 3 [2,31].

The current state-of-the-art in the field of soot oxidation and catalysis is characterized by several significant advancements [33,34]. Researchers have made substantial progress in the development of advanced catalysts, with a particular emphasis on ceria-based catalysts known for their high catalytic activity and oxygen storage capacity [35,36,37]. Previous studies have provided deeper insights into the underlying chemical processes involved in soot oxidation, shedding light on the role of catalyst materials and their surface properties [38,39]. A noteworthy trend is the exploration of synergistic catalysis, where combinations of catalyst materials are employed to enhance soot oxidation efficiency [40,41]. This approach has shown promise in lowering the operating temperatures required for effective soot removal. The practical applications of soot oxidation catalysts have diversified, extending beyond automotive exhaust systems to include industrial processes, diesel engines, and residential heating systems [42]. Additionally, stricter emission regulations and a focus on sustainability have further shaped the direction of research in this field, with an emphasis on developing eco-friendly and economically viable catalytic systems [43]. 

The methodology developed for soot oxidation represents a significant advancement in the field, introducing novel contributions that enhance our approach to pollution management and environmental protection [43,44]. Furthermore, the methodology pioneers the concept of synergistic catalysis, where multiple catalyst materials are combined to achieve remarkably improved catalytic performance, surpassing the capabilities of individual catalysts [13,45]. This innovative approach holds the potential to revolutionize the field by making soot removal more efficient and economical [46]. In addition to its catalytic advancements, the methodology delves into the mechanistic intricacies of soot oxidation [47]. Through comprehensive mechanistic studies, it unravels the underlying chemical processes, shedding light on reaction intermediates and pathways. This in-depth understanding enables the design of more targeted and effective catalysts, further advancing the field’s knowledge base [48]. Moreover, the methodology embodies sustainability by adhering to green chemistry principles [49]. It utilizes environmentally friendly and Earth-abundant materials in catalyst synthesis, reducing the environmental footprint associated with production and application [50]. This eco-conscious approach aligns with the growing emphasis on sustainability in scientific research. Unlike previous methodologies with narrow focuses, this approach recognizes the versatility of soot oxidation applications. It has been successfully applied not only in automotive exhaust systems but also in various industrial processes, diesel engines, and residential heating systems [51]. This broader application diversity highlights its real-world applicability and adaptability to different pollution management scenarios. Furthermore, the methodology is designed to ensure compliance with ever-evolving emission regulations [52]. By developing catalytic systems that not only meet but often exceed stringent standards, it equips industries and vehicles with the means to reduce their environmental impact while remaining within legal boundaries. 

## 2. Effect on Health and the Environment

Persistent uptake of diesel output materials could lead to various chronic breathing illnesses. Also, diesel exhaust is complex mixture; research indicates it has a harmful influence on human health and damages the green atmosphere [53]. The capital city of China (Beijing) is facing enormous problems with its air quality, with smog occurring very frequently [54]. Among all the contaminants that cause smog, a considerable part originates from locomotive dissipation. 

Soot particles in the atmosphere can absorb sunlight and heat, contributing to global warming [55]. This is particularly concerning in regions with snow and ice, as the presence of soot on these surfaces can cause them to absorb more heat, accelerating the melting process [56]. Soot can settle on surfaces, including vegetation and bodies of water. This can harm ecosystems and aquatic life. When soot settles on ice or snow, it reduces their reflectivity (albedo), causing more heat absorption and faster melting [57]. Exposure to soot particles has been linked to a range of health problems, including cardiovascular diseases, lung cancer, and premature death. Children, the elderly, and individuals with pre-existing health conditions are particularly vulnerable [58]. The main focus of this research project is on the control of diesel discharges, mainly to eliminate soot materials from diesel engines using various types of catalysts [59]. If the removal capacity of the catalyst is successfully attained and improved, it could possibly be used to decrease the emission of particles, increase air quality, and therefore improve living standards. China, as one of the largest countries with a massive population, has a significant influence on the lives of people inside and outside of the country [60]. Noxious carbonaceous soot materials, as well as other molecular complexes, are present in diesel. In the US and EU, the construction of preventive laws has led to regulations that decrease the discharge of soot.

**Figure 4:**
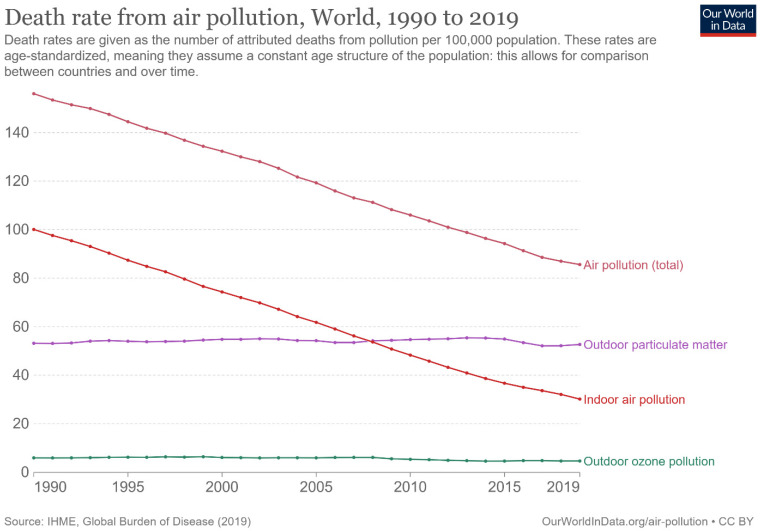
Death rate due to air pollution from 1990 to 2019 [63].

One of the most efficient techniques for removing soot particles is trapping them through filters, followed by oxidation. Meanwhile, the oxidation of diesel exhaust occurs at elevated temperatures (600 °C) with uncatalyzed soot filters [61]. Thus, a catalyst coating of the filters is needed to lower the soot oxidation temperatures to values within the temperature range of diesel exhausts (200–350 °C) [62]. Additionally, a catalyst is obligatory for soot removal; platinum-based catalysts are considered highly efficient, wherein NO reacts with oxygen to form NO_2_ in the dissipated gas. This has a substantial consequence for soot oxidation. However, platinum has the disadvantage of its high cost; hence, an inexpensive alternative is desirable. Soot has adversely affected human health for numerous years as described in Figure 4 [63]. Soot is a byproduct of burning fossil fuels for energy generation [64]. While it is crucial to reduce soot emissions to mitigate environmental and health impacts, fossil fuels still play a significant role in providing energy for many communities worldwide [65]. Transitioning to cleaner energy sources, such as renewables and natural gas, is essential to reduce soot emissions while ensuring a stable energy supply [66]. Similarly, some industrial processes, such as the production of steel and cement, may generate soot as a byproduct [67]. However, there is a growing emphasis on developing cleaner and more sustainable technologies to reduce soot emissions from these processes. In many parts of the world, especially in low-income countries, people rely on traditional biomass stoves and open fires for cooking and heating. These practices release a significant amount of soot. It is essential to address this issue by promoting cleaner and more efficient cooking and heating technologies to improve indoor air quality and reduce health risks. Furthermore, it is crucial to mitigate its production and emissions by transitioning to cleaner energy sources, adopting cleaner industrial processes, and promoting cleaner cooking and heating technologies while also addressing the energy and economic needs of communities worldwide.

## 3. Diversity in Catalysts for Removal of Soot

Recent research efforts have focused on developing platinum group metal-free catalysts for soot oxidation. Moreover, innovative catalyst materials, such as transition metal oxides and non-noble metal catalysts, have shown promising results in catalyzing soot oxidation while addressing cost and sustainability concerns [68]. Similarly, nanostructured catalyst materials have emerged as a prominent trend in soot oxidation research [69]. Nanostructured catalysts exhibit increased surface area and enhanced catalytic activity, enabling more efficient soot oxidation at lower temperatures. Research has explored various nanoarchitectures, including nanoparticles, nanowires, and nanotubes, to optimize catalytic performance [70,71]. Advances in catalyst support materials have further improved the durability and effectiveness of catalysts in soot oxidation [72,73]. Innovative support materials, such as perovskites, zeolites, and mesoporous materials, have been investigated for their potential to enhance catalyst stability and longevity under harsh operating conditions [74,75,76]. However, the identification of ceria-based catalysts as highly effective materials for soot oxidation has only recently been reported [40,77]. Ceria-based catalysts, known for their oxygen storage and release capabilities, have played a prominent role in reducing the temperature required for soot oxidation and enhancing catalytic activity. In this study, various types of catalysts of soot oxidation were reviewed [71]. 

**Figure 5:**
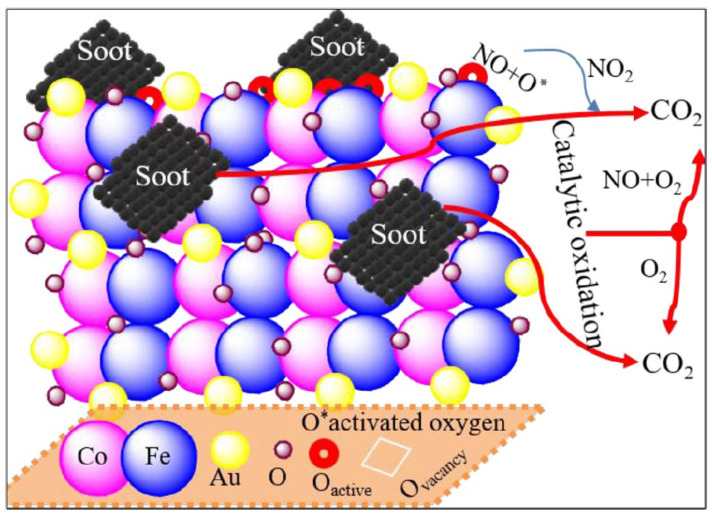
Mechanism of the Au–CoFe_2_O_4_ catalyst in soot oxidation [79].

PGM (platinum group metal) catalysts are extremely dynamic [78,79] (Figure 5). PGMs, including platinum, palladium, and rhodium, are commonly used as catalysts for the oxidation of soot in various applications, particularly automotive exhaust systems. The surface environments of the costly metals and catalysts, their surface area, and other surface parameters significantly clean the emissions gas during automobile catalytic conversion. Normally, Rh, Ru, Au, and Pt particles adhere to the base material. However, they are costly, and they are susceptible to greater price increases with increasing demands due to their low richness [80,81]. Consequently, efforts to find a catalyst free of PGMs or a catalyst with less noble metals are of universal importance. Recently, substantial research has been reported to improve the efficiency and low cost of PGM-free catalysts for diesel soot as well as NO*_x_* removal simultaneously [82,83]. To date, PGM-free compounds studied for soot oxidation [84] have been discussed based on their classification. 

**Figure 6:**
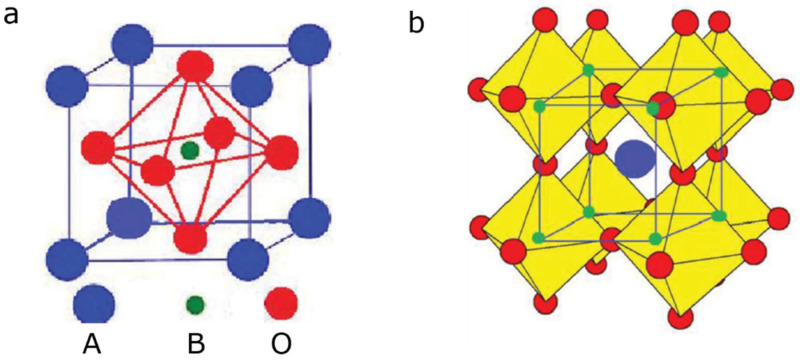
An ideal cubic perovskite structure of the proposed ABO_3_ formula (**a**) and its framework (**b**) [86,87].

Perovskite catalysts have gained significant attention in recent years due to their potential applications in various catalytic processes, including soot oxidation [85]. They are normally represented using the formula ABO_3_, wherein the A and B parts represent two cations of dissimilar dimensions, while O is an anion bridge that interacts with cations (Figure 6) [86,87]. Part A generally belongs to elements of alkaline/alkali earth metals (Sr, Cs, Ca, Ba, Ra) [88,89,90,91,92] and/or rare earth (La, Ce, Nd, etc.) [93,94,95] with a larger radius of approximately 0.90 Å compared with the transition metal of part B (Ag [96], Fe [97], Co, Zn, Cu, Ni, Mn, Cr, Ru, Al [94]) with an approximate radius of 0.51 Å [98]. In a cubic cell, the constituent A-atom occupies the dice corner positions (0, 0, 0), B conquers the position of body center (1/2, 1/2, 1/2), and O occupies the position of face center (1/2, 1/2, 0). In addition, part A, which is normally coordinated to 12 oxygen molecules, forms a dodecahedral site. Part B is occupied by six O-atoms in octahedral coordination [99,100]. In a proper formulation, a variety of metal ions of various valences could be substituted for A or B; therefore, many desirable properties could be obtained. Notable, parts A and B, as well as the valence state, determine perovskite catalytic properties [101,102].

**Figure 7:**
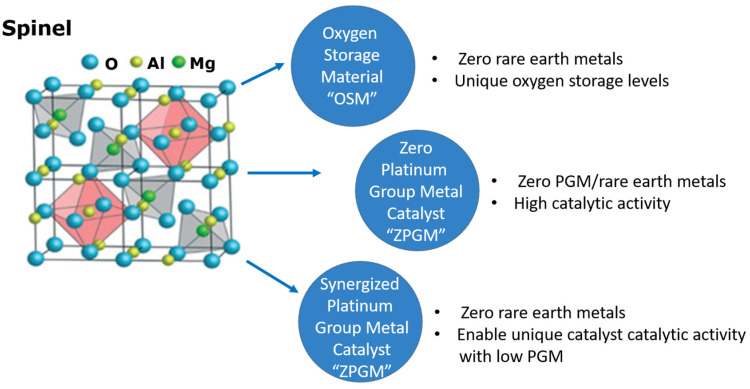
The spinal structure and its unique features [105,107].

Figure 6 depicts the general empirical structure of a typical spinal catalyst: M is a representative element from the IB, IIA, or IIB groups, and N is a representative metal (Group IA; zero < *x* ≤ 1; zero ≤ *y* ≤ 0.5; and zero < (*x* + 2*y*) ≤ 1). In these mathematical parameters, N and M metal atoms could be substituted for co-ions in the crystal frame, and during spinel formation, the abundance of N and M tries to separate non-spinel oxide phases, which are usually distributed throughout the spinel phase [103,104,105]. Moreover, heat generated through the exhaust gas produces particles that tend to agglomerate into larger particles [106]. Thereby, the surface area of these metal catalysts is reduced, resulting in a decline in their catalytic performances (Figure 7) [12,107].

**Figure 8:**
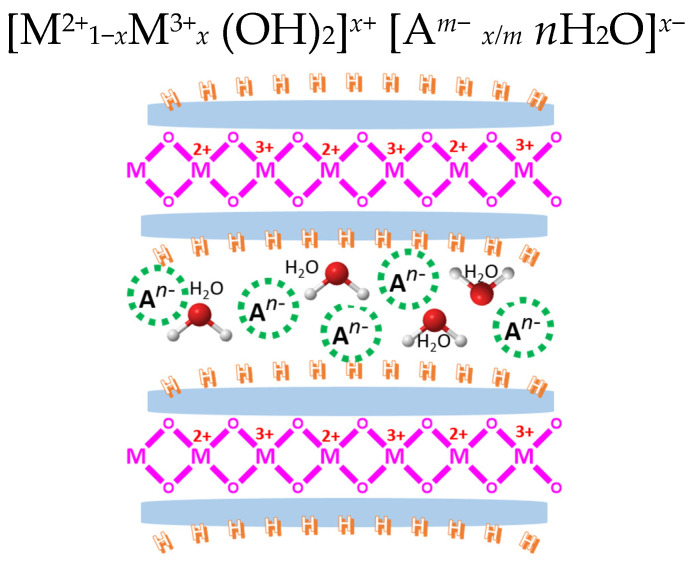
The proposed structure of layered double hydroxides with a presenting anion and cation framework; image regenerated from [111].

Layered double hydroxides (LDHs) are a class of materials that have been explored for various catalytic applications, including soot oxidation. LDHs are also known as hydrotalcite-like compounds or anionic clays. They are made of positively charged metal hydroxide layers and charge-balancing anions in the interlayer regions [108,109], as displayed in Figure 8, with the following general formula and structure [110,111]. Their tunable composition and surface properties make them promising candidates for catalytic reactions, including the oxidation of soot (carbonaceous particulate matter) [112,113]. 

**Figure 9:**
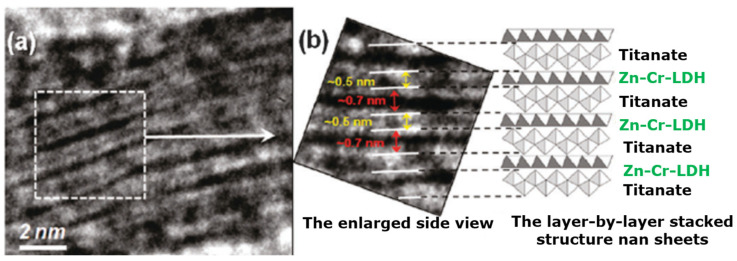
HR-TEM image of the layered structure of ZnCr-LDH (**a**) and a enlarged image showing the structural system (**b**) [114].

**Figure 10:**
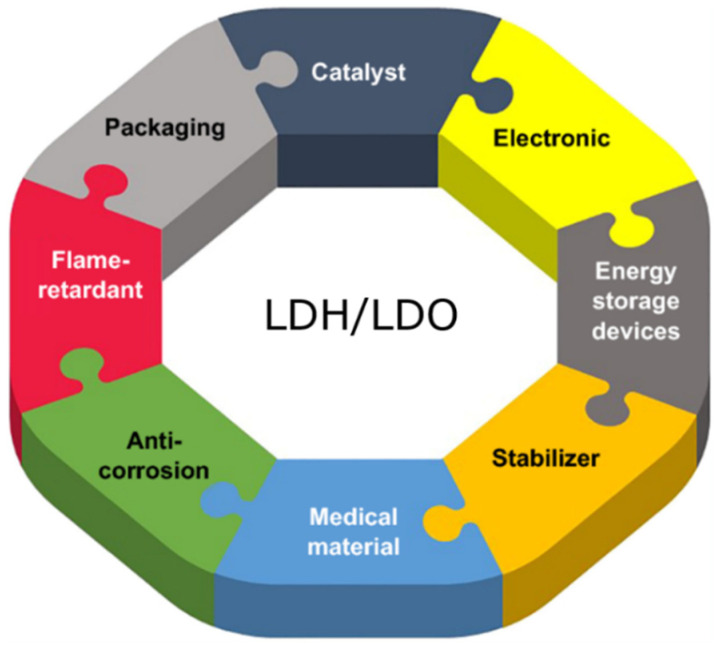
Chart of the various applications of LDH and LDO materials [121,122].

LDHs exhibit anion exchange characteristics due to the weakly carbonate-bonded anions in their interlayer region. Moreover, selection for the anions can be done at the initial precipitation during its preparation. It is important to mention here that during calcination, metal-oxide products can be produced from these LDHs, which are further used for various applications [114,115]. In addition, the resultant metal oxides possess higher surface areas, which is normally beneficial for catalysis applications. An HRTEM image of the layered framework of ZnCr-LDH is revealed in Figure 9 [114,116,117]. Layered double oxides (LDOs) are a class of materials that share some similarities with layered double hydroxides (LDHs) but are composed of metal cations and oxygen anions arranged in a layered structure [118,119]. These materials have also been investigated in various catalytic applications, including soot oxidation. LDOs can be tailored to have specific compositions and structures to optimize their catalytic activity in the oxidation of carbonaceous soot particles [112,120]. Various applications for LDH and LDO are shown in Figure 10 [121,122]. 

**Figure 11:**
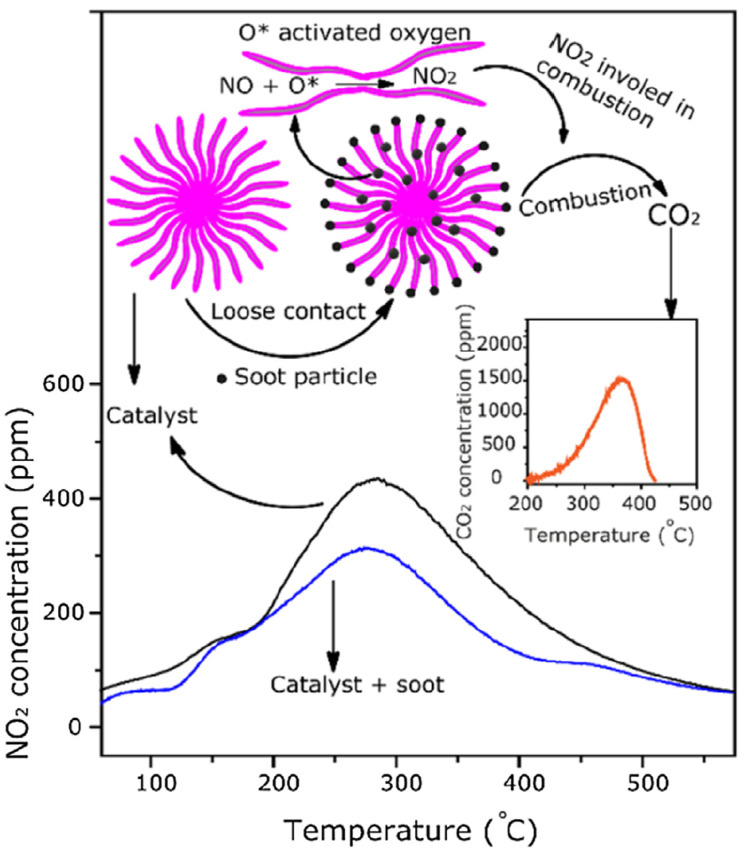
A graphical explanation of the role of mixed metal oxides materials with NO in soot oxidation [115].

Mixed metal oxides (MMOs) of inner and outer transition metals, alkaline, rare earth, and alkali group metals have potential for various catalytic applications [123]. Different reactions have been used for MMO synthesis. For example, alkylation, the Mannich reaction, oxidation, reduction, multicomponent, condensation, cycloaddition, deprotection, hydroxylation, and other reactions can be done successfully in different reaction conditions [124]. The mixed metal oxides use an interesting mechanistic approach to convert the NO and soot into their respective components, such as NO_2_ and CO_2_ (Figure 11) [115]. 

## 4. Ceria-Based Mixed Metal Oxides

Ceria (CeO_2_) is of most significant importance as a component of three-way catalysts (TWCs) given its storage capacity (OSC) for oxygen [125]. It has attained a significant rank among the metal oxides that have been extensively studied to date [126,127]. The research direction proposed by Trovarelli has opened a new door for ceria-based catalysts, indicating their potential in theoretical and practical applications as well as providing structural insights for their derived catalysts. Meanwhile, they function to support and boost the catalytic performances of metal catalysts [128]. The effects of the nanometric sizes and morphologies of ceria-based catalysts have been studied since the last decade, and various studies have reported on their synthesis pathways, chemical properties, geometries, characteristics, and catalytic performance in the oxidation of CO to date [129]. Recently, a correlation has been reported for redox properties between surface properties and the crystal morphology of ceria-based cubes, polyhedrons, and rods. Observations indicated that face reconstruction, size, and nanomorphology influence their performance, selectivity and stability [130]. In the ceria cubic structure, the fcc group, which is regarded as a stable surface plane, shows a lower coordination number compared to its bulk with divergent terminating structures on surfaces, including repetitive O-Ce-O interlayers, both elements Ce and O, and a O-Ce-O-Ce echoing unit. However, in thermally controlled systems, stable surfaces are normally generated during crystal growth and finally develop specific nanoshapes [131]. However, by changing synthesis parameters (for example, reaction system pH, precursor nature, pressure of the solvent, etc.), the growth rate can be controlled in various directions. Meanwhile, solvothermal and/or hydrothermal growth processes are template-free, prominent methods used to develop various shapes at the nanoscale [132].

**Figure 12:**
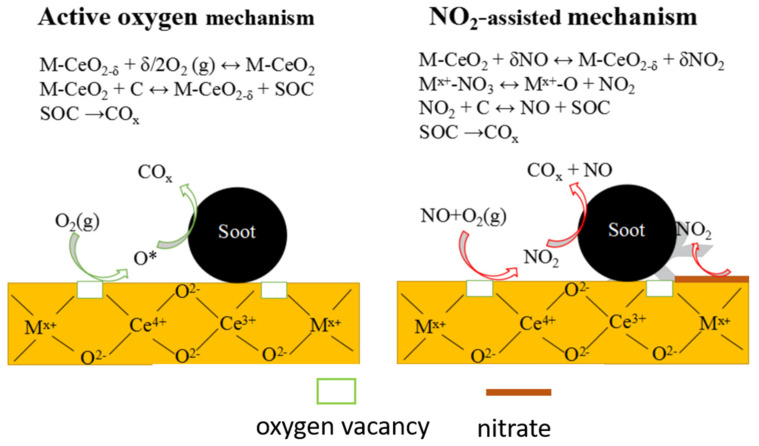
Possible soot oxidation paths on cerium based catalysts [136].

Remarkably, every stable plane displays various reduction features. The redox process of Ce^4+^ to Ce^3+^ produces vacancies for oxygen that play a vital role in oxygen packing and oxidation reactions. There is no theoretical basis; the growth plans follow the order of reactivity for oxygen vacancy defect formation, providing the basis for experimental work to assess the relationship between the catalytic performance and nanocrystal morphology of ceria [133]. The oxygen vacancies and surface chemistry strongly depend on the nanometric size of particles, and these factors are strongly enhanced when the particle size is less than 10 nm. Oxygen vacancy creation modeling investigations focused on size revealed that their energy is governed by the position of the oxygen atom lattice; for nanoparticles (NPs) with a size of 2–4 nm, its value approaches the minimum level [134]. Consequently, the catalytic activities and OSC are chiefly dependent on NP morphology; specifically, for nanoshapes, these features depend on various proportions of surface-terminating planes. It is well known that ceria rods, cubes, and octahedrons are enclosed with crystal planes. In addition, each category shows distinct oxygen vacancy content as a function of morphology and the structures associated with the exposed crystal planes and surface compositions. The decreasing trend in turnover frequency (TOF) for CO oxidation was observed in accordance with the order of reactive vacancy formation [135]. Figure 12 illustrates the potential pathways involved in the oxidation of soot on catalysts containing cerium (Ce) [136].

The catalytic performance of the nanorods was observed to be associated with loosely bound oxygen. The nanorods’ performances were lower than those of nanowires, regardless of the fact that nanorods and nanowires exhibit predominantly reactive planes; this could be attributed to a higher concentration of surface-active planes [137]. Hierarchically, mesoporous ceria is prepared using diatom templates, which have greater Ce^3+^ content, a high specific surface area (SSA) (78 m^2^ g^1^), facile reducibility, a higher number of oxygen vacancies, and enhanced CO oxidation compared with bulk ceria. Moreover, ultrasound synthesis was reported to form nanoflowers, nanospheres, nanorods, and nanoribbons of ceria nanostructures (size ~5 nm) [138]. This synthesis was performed in a single step using various kinds of ionic liquids. The shape and structure of the final product depend on how it was heated. For example, under [C4mim][Tf2N], the ionothermal fabrication method produced flower and nanorod shapes, while the ultrasound method produced nanospheres. Nanoshape activity order followed the order of the SSAs; however, this order was not found to be proportional to them, indicating that oxygen vacancies as well as structural defects play crucial roles. Sonochemistry under [C4mim][Tf2N] generates nanospheres with the best performance. This is because the nanospheres have a large SSA, a mesoporous structure, a higher number of surface oxygen vacancies, and small particle size [139]. Also, the hierarchical layer-stacking morphology was made using a sol-gel synthesis protocol that did not use a template. The morphology was flower- or spindle-like and composed of several porous nanoflakes with a size of approximately 10 nm. CO oxidation activity was found to be greater compared with its commercially available bulk counterpart, which is attributed to its extraordinary SSA (171.6 m^2^ g^−1^) [139,140].

Cerium-based mixed metal oxide catalysts are widely synthesized using the co-precipitation method. The fabrications generated through the co-precipitation method involve metal-soluble salts in the appropriate solvent systems followed by co-precipitation through the addition of either acid or base as well as some reagents that initiate precipitation. The perovskite catalysts could be readily transformed into their oxides by thermal treatments. The whole precipitation process is completed in three steps: (1) supersaturation, (2) nucleation, and (3) growth. Crystalline or an amorphous precipitate or gel is acquired that is subsequently aged, undergoes filtration, and is finally washed unless it is salt free. After the successful completion of these steps, further steps are taken: (1) drying, (2) shaping, (3) calcination, and finally (4) activation [141]. The coprecipitation process was then supported by a number of characterizations, which led to the conclusion that this method makes products with a higher surface area and less crystallinity [142]. Furthermore, the final product acquired through alkali coprecipitation pathways is depreciated by the alkaline metal impurities and through the formation of environmental trash (e.g., salts and washed water) [143]. Moreover, nanoceria-based catalysts play a pivotal role in reducing these emissions [144]. They facilitate the oxidation of soot particles, converting them into less harmful byproducts, such as carbon dioxide and water [145]. This process not only lowers the emission of toxic and carcinogenic compounds but also contributes to improved air quality in urban areas and regions with heavy diesel engine use. Additionally, it mitigates environmental damage caused by soot deposition on surfaces [146]. These catalysts help diesel engine manufacturers and operators comply with stringent emissions regulations, all while potentially enhancing engine efficiency and reducing maintenance costs due to their durability and longevity.

## 5. Mechanistic Chemistry for Removal of Soot 

For the catalytic oxidation or elimination of soot contaminants, a worthy appreciation of the essential reaction mechanism, functional scheme, and appropriate device configuration is very essential. These parameters might be helpful for synthesizing efficient catalysts with desired characteristics while planning the procedures and strategies for automobile discharge control. The novel idea of concurrent catalytic elimination of NO*_x_* and soot was reported for the first time [147]. Subsequently, several mechanisms have been reported [148]. Nevertheless, it is generally assumed that the reactions involved in these processes might be triggered by two mechanistic behaviors [149]. In the first stage of the process, it is conceivable that the catalyst’s surfaces may produce two adjacent oxygen vacancies through the burning of soot. Subsequently, these vacancies serve as active sites for the chemisorption of two NO molecules. In the interim, the NO that has been adsorbed undergoes detachment, resulting in the formation of N(ads) and O(ads), which eventually leads to the generation of N_2_O or N_2_. However, to date, the constructive effect of oxygen could not be elucidated, as oxygen could possibly complete the oxygen vacancies on the catalyst, therefore reducing the reaction speed.

The potential positive impact of oxygen was examined in depth, with the expectation of alternative mechanisms. In the second approach, the formation of C [N, O] was elucidated through the combustion of mercurial free-carbon (Cf) and NO species that had been adsorbed onto the catalyst’s surface and exhibited noteworthy properties. There exist two potentially viable pathways for the formation of N_2_. In the first pathway, the C [N, O] species undergoes a direct reaction with either another NO molecule or a gaseous NO molecule. In contrast, the second pathway changes the C [N, O] species into the C [N, N] species, a different reaction intermediate, which then breaks down into N_2_. The formation of active sites on the surfaces of carbon fibers (Cf) during the combustion of carbon can explain the increase in the generation of oxygen. These pathways facilitate the process of chemisorption by enhancing the likelihood of NO uptake. Another significant issue is associated with the facile production of nitrogen dioxide (NO_2_), which demonstrates greater reactivity in comparison to nitric oxide (NO). [150]. A crucial inquiry is the practical implementation of the aforementioned concept in real-world diesel engines, primarily owing to the diminished low-temperature reactivity of the catalysts. The typical range for the temperature of diesel engine exhaust is between 150 and 400 °C. However, the efficacy of NO*_x_* reduction measures for soot oxidation is generally limited while operating at temperatures below 200 °C. Due to its superior oxidative capabilities in comparison to O_2_, NO_2_ has a propensity for oxidizing soot at lower temperatures. Consequently, contemporary technologies predominantly rely on the reaction between carbon and nitrogen dioxide (C-N_2_O) as a primary mechanism [151]. The initial technological advancement in the field of NO*_x_* and soot catalytic reduction is commonly referred to as the continuous regenerating trap (CRT). The hypothesized mechanism is described as follows: The oxidation of NO occurs on the catalyst surfaces, which are connected upstream to the wall-flow monolith diesel filter. Simultaneously, the created NO_2_ reacts with the soot accumulated on the filter, resulting in the formation of NO and CO_2_. One of the primary challenges associated with the use of CRT technology is the permeation of NO_2_ through the filter surfaces, resulting in a reduced overall efficiency in eliminating total NO*_x_* [152].

**Figure 13:**
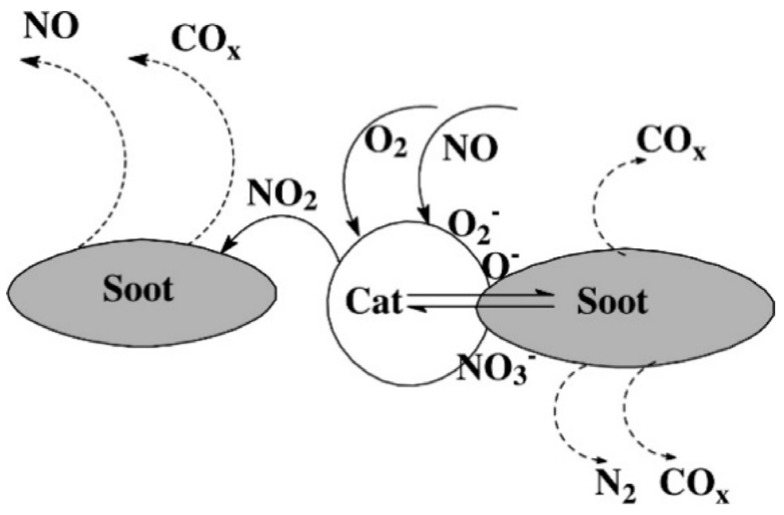
Mechanistic approach for soot oxidation in NO + O_2_ atmospheres [156].

Therefore, Toyota has introduced a novel diesel particulate-NO*_x_* reduction (DPNR) technology as a potential solution for the concurrent elimination of catalytic NO*_x_* and soot. The NSR catalyst was applied to the interior pore surfaces and cell walls of the diesel particulate filter (DPF) substrate. In situations characterized by strong muscular tension, the compound NO may undergo oxidation to create NO_2_. This process occurs concurrently with the activation of molecular oxygen, which results in the adsorption of oxygen atoms on Pt-active sites. During this period, a significant portion of the NO_2_ is converted into nitrate or nitrite using a storage catalyst. Furthermore, the residual activated oxygen atoms and NO_2_ molecules have the potential to engage in a direct reaction with diesel soot. Upon the enrichment of the atmosphere, the nitrate or nitrite species, or both, undergo decomposition on the surfaces of NO_2_. Through the action of soot, CO, and HC at Pt-active sites, this process of breaking down would then turn NO_2_ into N_2_ [153]. Therefore, it can be readily inferred that the implementation of a suitable operational strategy and device arrangement is crucial in achieving a remarkable reduction in both NOx and soot emissions. There is a significant demand for a multifunctional catalyst that can effectively oxidize NO to NO_2_ and simultaneously store NO_x_ under lean conditions. Additionally, this catalyst should exhibit selective reduction of NO*_x_* to N_2_ during the NO_2_ soot process [154,155], and the mechanism is proposed in Figure 13 [156]. 

## 6. Conclusions, Future Prospects, and Challenges

In this comprehensive review, we have not only provided an overview of the introduction of soot and its adverse impacts on both human health and the environment but also detailed the various catalysts involved in the process of soot oxidation, with a particular focus on ceria-based mixed metal oxide catalysts. Furthermore, we have explored the functionality of different types of catalysts, including platinum group metals, perovskite, mixed metal oxides, layered double hydroxides, and layered double oxides, in the context of soot removal. Additionally, we have delved into the underlying mechanistic chemistry governing the soot oxidation process, highlighted future prospects in this field, and critically examined the challenges associated with effective soot oxidation. In summary, our findings lead to several key conclusions: (1) Soot emerges as an inevitable by-product during the combustion of hydrocarbon fuels. (2) Soot particles, primarily emitted by automobile engines, pose significant threats to the environment and human health, contributing to problems, such as photochemical smog and the development of lung cancer. (3) The pursuit of highly efficient catalysts for the removal of soot has garnered increasing attention due to its importance. (4) We have reviewed the mechanisms underlying soot formation in modern vehicle engines in diverse conditions and have comprehensively assessed methods for reducing soot emissions. (5) Our review serves as a valuable reference for researchers studying the characteristics of oxygenated fuels and exploring strategies to mitigate soot emissions, thereby laying a solid foundation for further investigations in this field.

Regarding the future prospects and challenges, key areas of future focus and potential developments are noted as follows: (a) The future of soot oxidation catalysis lies in the precise tailoring of catalyst materials at the nanoscale. Researchers will explore innovative approaches to design catalysts with optimized composition, crystal structure, and morphology to achieve unparalleled catalytic activity. These tailored catalysts will be engineered for specific applications, ensuring maximum efficiency and reduced energy consumption. (b) Expectations are high for the development of multifunctional catalysts capable of addressing multiple pollutants simultaneously. This integrated approach will allow for the removal of not only soot but also other harmful emissions, such as nitrogen oxides (NO*_x_*) and volatile organic compounds (VOCs). The design of catalysts that can tackle complex mixtures of pollutants will be a pivotal area of research. (c) The exploration of synergistic interactions between different catalyst materials and co-catalysis systems will continue to expand. Combining various catalysts to enhance overall catalytic performance and selectivity will be a focal point. Researchers will seek to better understand the mechanisms governing these interactions and leverage them for more efficient soot oxidation. (d) The development and utilization of advanced characterization techniques, such as operando spectroscopy and in situ microscopy, will become increasingly prevalent. These techniques offer real-time insights into catalyst behavior during soot oxidation, providing a deeper understanding of reaction mechanisms and catalyst deactivation processes. (e) Sustainability will be a driving force in future research. Green chemistry principles will guide the synthesis of catalysts, emphasizing the use of Earth-abundant and environmentally friendly materials. Researchers will strive to minimize the environmental impact of catalyst production and operation while maximizing performance. (f) Heterogeneous catalysis for soot oxidation will find new and diverse applications beyond traditional sectors. These may include the development of catalysts for emerging energy technologies, green chemistry processes, and novel environmental remediation solutions. (g) As emission regulations become more stringent globally, there will be a growing need for catalysts that not only meet but exceed these standards. Industry adoption of advanced soot oxidation catalysts will increase, driving commercialization and the integration of these technologies into various sectors. (h) With increasing urbanization, the focus on public health and urban air quality will intensify. Soot oxidation research will play a crucial role in reducing particulate matter emissions and improving air quality in densely populated areas. (i) Future research in soot oxidation and catalysis will benefit from interdisciplinary collaboration among chemists, materials scientists, engineers, environmental scientists, and policy makers. These collaborations will facilitate the development of holistic solutions to complex pollution challenges.

Despite the tremendous progress made in soot oxidation catalysts, there are still obstacles to overcome in developing highly effective soot oxidation catalysts, such as a thorough investigation of catalyst deactivation by other components in exhaust (sulfur, phosphorus and water vapors) and their possible regeneration during application. On the catalytic material side, new challenges should be addressed regarding the (1) synthesis of multi-component catalysts using a combinatorial approach and (2) controlled catalyst morphology using novel synthesis techniques for desirable physio-chemical properties. Future generations of scientists and engineers should continue this trend in the development of advanced soot oxidation catalysts with both high activity and stability.

## Figures and Tables

**Table 1:** Threats of exhaust components to human life [9,12].

Pollutants	Concentration	Threats
Soot	20–200 mg/m^3^	Eyes problems, cancer, asthma, skin infections, lung damage, heart issues
NO*_x_*	30–1000 ppm	Chest pain, respiratory and lungs problems, cough
SO*_x_*	Proportional to fuel S content	Acid rain, skin problems
CO_2_	2–12 vol%	Green house effect, acid rain, lung disease
CO	100–1000 ppm	Hpertension, head pressure, lung disease
HC	50–500 ppm	Eyes irritation, lungs issues, respiratory problems
PAH	0.3 mg/mil	Kindney and liver damage

## Data Availability

The data will be available upon request.
